# Rationalisation of meat consumption in New Zealand adolescents

**DOI:** 10.1017/S1368980021003244

**Published:** 2022-04

**Authors:** Kelly R Latimer, Meredith C Peddie, Tessa Scott, Jillian J Haszard

**Affiliations:** 1 Department of Human Nutrition, University of Otago, Dunedin, New Zealand; 2 Division of Sciences, University of Otago, P.O. Box 56, Dunedin 9054, New Zealand

**Keywords:** Meat consumption, Rationalisation, Vegetarian, Adolescent

## Abstract

**Objective:**

This study aimed to describe meat consumption rationalisation and relationships with meat consumption patterns and food choice motivations in New Zealand adolescents.

**Design:**

This was a cross-sectional study of adolescents from high schools across New Zealand. Demographics, dietary habits, and motivations and attitudes towards food were assessed by online questionnaire and anthropometric measurements taken by researchers. The 4Ns questionnaire assessed meat consumption rationalisation with four subscales: ‘Nice’, ‘Normal’, ‘Necessary’ and ‘Natural’.

**Setting:**

Nineteen secondary schools from eight regions in New Zealand, with some purposive sampling of adolescent vegetarians in Otago, New Zealand.

**Participants:**

Questionnaires were completed by 385 non-vegetarian and vegetarian (self-identified) adolescents.

**Results:**

A majority of non-vegetarian adolescents agreed that consuming meat was ‘nice’ (65 %), but fewer agreed that meat consumption was ‘necessary’ (51 %). Males agreed more strongly than females with all 4N subscales. High meat consumers were more likely to agree than to disagree that meat consumption was nice, normal, necessary and natural, and vegetarians tended to disagree with all rationalisations. Adolescent non-vegetarians whose food choice was motivated more by convenience, sensory appeal, price and familiarity tended to agree more with all 4N subscales, whereas adolescents motivated by animal welfare and environmental concerns were less likely to agree.

**Conclusions:**

To promote a reduction in meat consumption in adolescents, approaches will need to overcome beliefs that meat consumption is nice, normal, necessary and natural.

The consumption of meat has attracted considerable attention in recent years^(1)^ due to numerous negative effects of meat production and consumption on both population and planetary health^(1–3)^. Research has highlighted strong correlations between consumption of red and processed meats and the risk of developing non-communicable diseases, such as CVD, type 2 diabetes and colorectal cancer^(4–6)^. Increasing awareness of climate change has also raised concerns surrounding the sustainability of meat production and the short- and long-term effects on our environment^(2,7)^. Alongside these concerns, ethical and animal welfare concerns have been raised, because the increasing industrialisation of meat production has caused a substantial deterioration in welfare^(2)^.

Experts are therefore calling for a global shift to a plant-based diet, defined as the habitual increased consumption of fruits, vegetables, legumes, nuts, seeds and a variety of grains (including wholegrains), alongside the decreased or minimal consumption of meat, eggs and dairy products^(8)^. It is hypothesised that a shift to a plant-based diet would be beneficial in terms of reducing the risk of non-communicable diseases, reducing the unethical treatment of animals and maintaining environmental sustainability^(7,9–11)^. A Summary Report of the EAT-Lancet Commission^(10)^ identified that the consumption of fruits, vegetables, legumes and nuts needed to more than double, while the consumption of red meat needed to more than halve in order to meet their Scientific Targets for Healthy Diets and Sustainable Food Production by 2050.

To be able to develop consumer-orientated strategies for a transition to a more plant-based diet, personal determinants of behaviour, including habits, attitudes, knowledge and barriers to change, must be recognised^(12)^. One key barrier to changing meat consumption habits in the population will be overcoming the rationalisations that people make when consuming meat. Many omnivores face a ‘meat paradox’ where they enjoy consuming meat but do not like the thought of harming animals or the thought that their meat consumption is contributing to global warming^(3,13)^. Therefore, consumers rationalise their meat consumption by contending that meat is *natural* – part of human nature and biology; *necessary* – one cannot live without meat, and nutrients found only in meat are essential for a healthy, balanced diet; *normal* – how we behave based on customs or traditions; or *nice* – the taste of meat and associated satisfaction^(3,14)^. These rationalisations are referred to as the ‘4Ns’, with research in adults showing that these four concepts cover the majority of rationalisations when it comes to meat consumption^(14)^. The 4Ns enable omnivores to continue to eat meat with low levels of guilt and motivation to change meat-eating behaviours^(14)^. Therefore, to overcome a commitment to meat consumption, these rationalisations need to be addressed.

Adolescence presents an opportunity to modify meat consumption at a stage in life that sees an increase in autonomy surrounding food choices^(15)^, as well as the development and increasing awareness of personal values. Furthermore, dietary habits developed during this time may track into adulthood^(16)^, meaning that interventions or public health messages targeting the adolescent diet may create long-term improvements in population health and planetary outcomes. Previous research suggests adolescents tend to be ‘present-oriented’ and are less concerned about long-term health issues, when compared to older adults^(17,18)^. Therefore, if behaviour change strategies are to be effective, an understanding of adolescent motivations for food choice and rationalisation of meat consumption is crucial for health promotion, informing policy and food manufacturers, as well as others who hold an interest in promoting a population shift to a more plant-based diet^(17)^. Using the 4Ns questionnaire, the present study aimed to describe meat consumption rationalisation and relationships with meat consumption patterns and food choice motivations in a sample of New Zealand adolescents.

## Methods

### Study design

The present study is the secondary analysis of the Survey of Nutrition, Dietary Assessment and Lifestyles (SuNDiAL) project, which encompasses two New Zealand-wide cross-sectional surveys, one of female adolescents conducted between February and October 2019 and the other of male adolescents conducted between February and April 2020 (all data for this analysis were collected prior to COVID-19 lockdown). The project was registered with the Australian New Zealand Clinical Trials Registry: ACTRN12619000290190 (2019) and ACTRN12620000185965 (2020).

### Participants and recruitment

Recruitment of the girls has been described in detail previously^(19)^, with similar procedures followed for recruitment of the boys. Briefly, secondary schools were recruited by email in locations where data collectors were based. A total of nineteen secondary schools from eight regions in New Zealand were recruited across the two studies – thirteen schools for the female cohort in 2019 and six different schools for the male cohort in 2020. The number of schools for the male cohort was considerably lower than for the female cohort because recruitment was cut short in 2020 due to COVID-19 lockdown. Data collectors then gave presentations to each of the recruited schools as part of whole-school assemblies, or to year groups or individual classes, to initiate adolescent recruitment. Interested and eligible participants were given a study information sheet and invited to sign up via email or via the study website. In addition to recruitment through high schools, there was purposive sampling of vegetarians in the 2019 female cohort to meet the primary objective of that phase of the SuNDiAL project, which was to compare the dietary intakes of vegetarian and non-vegetarian female adolescents^(19)^. This included targeted recruitment advertisements through Facebook in Dunedin and Christchurch of vegetarians not from recruited schools. Participants were eligible to participate if they self-identified as either male (in 2020) or female (not pregnant in 2019), were between 15 and 18 years of age, and spoke and understood English.

### Data collection

Once online consent was obtained, participants were given a series of online questionnaires to complete in their own time. These included questions about demographics, health, dietary habits, and motivations and attitudes towards food and were administered online through REDCap (Research Electronic Data Capture)^(20)^. The data collectors visited the schools to collect anthropometric measurements (height and weight), assign accelerometers to participants for the measurement of physical activity and conduct the first of two 24-h diet recalls (the second being conducted during a follow-up phone or video call within the following 2 weeks). The analysis presented here only utilises data from the online questionnaires and the anthropometric assessments.

### The 4Ns questionnaire

The 4Ns questionnaire consists of sixteen statements about different types of rationalisations when it comes to meat consumption^(14)^. There are four subscales (four items per subscale), scored on a seven-point scale: participants were asked to mark how strongly they agreed or disagreed with the given statements. The subscales are *Natural* (‘it is only natural to eat meat’; ‘it is unnatural to eat an all plant-based diet’; ‘our human ancestors ate meat all the time’; ‘human beings naturally crave meat’), *Normal* (‘not eating meat is socially unacceptable’; ‘it is abnormal for humans not to eat meat’; ‘it is normal to eat meat’; ‘most people I know eat meat’), *Necessary* (‘it is necessary to eat meat in order to be healthy’; ‘you cannot get all the protein, vitamins and minerals you need on an all plant-based diet’; ‘human beings need to eat meat’; ‘a healthy diet requires at least some meat’) and *Nice* (‘meat adds so much flavour to a meal it does not make sense to leave it out’; ‘the best tasting food is normally a meat based dish (e.g. steak, chicken breast, grilled fish)’; ‘meals without meat would just be bland and boring’; ‘meat is delicious’). Scores for each subscale were calculated as the mean of all items, giving a score between 1 (strongly disagree) and 7 (strongly agree). Agreement with the rationalisations (subscales) was categorised based on a score of greater than 4.

### Household deprivation

Participants provided their home address which was used to determine their New Zealand Deprivation Index 2018 decile^(21)^. This was then collapsed into three categories: low deprivation (deciles 1 to 3); medium deprivation (deciles 4 to 7) and high deprivation (deciles 8 to 10).

### BMI

Height and weight were measured using standard protocols^(19)^. BMI was calculated and *Z*-scores determined using the WHO growth charts^(22)^. Overweight was classified as those with a *Z*-score greater than 1 and less than or equal to 2; and obese was classified as those with a *Z*-score greater than 2.

### Meat consumption habits

Participants were asked at the beginning of the online survey ‘Are you vegetarian or vegan?’ with yes/no options. Meat consumption habits were assessed using the Dietary Habits Questionnaire^(23)^. Participants were asked how often they consume processed meats, other red meats, pork, poultry, fish or other seafoods, with answer options including ‘more than 3 times a day; 2–3 times a day; once a day; 5–6 times a week; 2–4 times a week; once a week; 2–3 times a month; monthly; rarely or I do not eat these’. These meat variables were combined to give an overall ‘number of times a week’ meat was consumed. This was then collapsed into a binary variable: ‘Low meat consumers’ (no more than five times a week) and ‘Moderate/high meat consumers’ (more than five times a week).

### Food choice motivations

Motivations for food choice were measured using a combination of the Food Choice Questionnaire^(24)^ and the Ethical Food Choice Motives Questionnaire^(25)^. These questionnaires have been well validated in several populations, including in a sample of Irish adolescents^(17,24–26)^, and were specifically developed to assess both health-related and non-health-related factors that affect food choice, as well as factors relevant to vegetarians (e.g. animal welfare, environmental protection and religion). Participants were asked how important the food they eat on a typical day resonated with the given statement. Food choice motivations were measured on a scale from 1 to 4 (1 = not at all important; 4 = very important). Eight of the nine subscales from the Food Choice Questionniare were used, with the ‘Ethical concern’ subscale replaced by the ‘Environmental concerns’, ‘Animal welfare’ and ‘Religion’ subscales from the Ethical Food Choice Motives questionnaire^(25)^. In total, the eleven subscales were *Health (6 items)*, *Mood (6 items), Convenience (4 items)*, *Sensory appeal (4 items)*, *Natural content (3 items)*, *Price (3 items)*, *Weight control (3 items)*, *Familiarity (3 items), Animal welfare (2 items), Environmental concerns (3 items)* and *Religion (2 items)*. Subscale scores were calculated as the mean of all items.

### Statistical methods

All statistical analyses were undertaken in Stata 16.1 (StataCorp.). To determine demographic predictors of the meat rationalisation scores in non-vegetarians, mixed effects regression models were used, with the subscale score as the dependent variable and the demographic variable (sex, age, deprivation, BMI *Z*-score or weight status) as the independent variable. School was included as a random effect. Mean differences, 95 % CI and *P*-values were calculated.

To illustrate the differences in the distributions of the meat rationalisation subscale scores between non-vegetarians and vegetarians, box plots were generated. The relationships between meat consumption patterns (low consumers *v*. moderate/high consumers) and meat consumption rationalisation scores were assessed using mixed effects regression models as before, but with further adjustment for age, sex and deprivation. To assess correlations between meat consumption rationalisation scores and food choice motivations, correlation coefficients were calculated for non-vegetarians. Residuals of all regression models were plotted and visually assessed for homogeneity of variance and normality.

## Results

### School recruitment and participant response

Thirteen secondary schools consented to participate and were recruited in 2019, across which 257 females consented to participate. Fifteen vegetarian females were additionally recruited in 2019 through targeted advertising. Complete data for this assessment were collected from a total of 259 female participants. Eight secondary schools consented to participate and were recruited in 2020; however, due to the restrictions in place with COVID-19 during 2020, only six of the schools were able to participate. One hundred and forty-six males consented to participate, with data collected from a final sample of 126 male participants. Overall, 385 participants enrolled, consented and provided data for this secondary analysis of the SuNDiAL project.

### Demographic characteristics of participants

Forty-eight (12·5 %) participants self-identified as vegetarian, forty-four (91·7 %) of whom were female. Vegetarians tended to be slightly older, live in an area of lower deprivation and be of a healthy weight; however, the proportions of participants with low, medium and high levels of deprivation and who were categorised as healthy, overweight or obese were similar in non-vegetarian males *v*. females (Table 1).

**Table 1:** Demographic and anthropometric characteristics of all participants (*n* 385)

	All non-vegetarians		Male non-vegetarians		Female non-vegetarians		Vegetarians*	
	*n* 337		*n* 122		*n* 215		*n* 48	
	*n*	%	*n*	%	*n*	%	*n*	%
Age, years								
Mean	16·7		16·6		16·7		17·1	
sd	0·8		0·7		0·9		0·8	
Household deprivation†								
Low	124	37·0	42	34·7	82	38·3	22	45·8
Medium	143	42·7	52	43·0	91	42·5	17	35·4
High	68	20·3	27	22·3	41	19·2	9	18·8
BMI *Z*-score‡								
Mean	0·63		0·43		0·73		0·31	
sd	1·05		1·16		0·97		0·91	
Weight status‡	292	86·6	98	80·3	194	90·2	41	85·4
Healthy weight	191	65·4	64	65·3	127	65·5	33	80·5
Overweight	75	25·7	28	28·6	47	24·2	5	12·2
Obese	26	8·9	6	6·1	20	10·3	3	7·3

* Fifteen female vegetarians were recruited through targeted methods; 44 (91.7 %) of the vegetarians were female. Vegetarians were self-identified.

† Household deprivation measured using deciles of the NZDep2018 index, with Low: deciles 1–3; Medium: deciles 4–7; High: deciles 8–10. Two participants in the non-vegetarian sample were missing these data.

‡ BMI Z-score calculated using the WHO growth charts. Overweight: BMI Z-score ≥1 & <2; Obese: BMI Z-score ≥2. 52 participants did not have anthropometric measures undertaken (*n* 45 non-vegetarians and *n* 7 vegetarians).

### Predictors of meat consumption rationalisation in non-vegetarains

Table 2 reports mean differences (95 % CI) in the 4Ns subscale scores between different demographic groups (i.e. sex, household deprivation and weight status), as well as for age (for each year older) and BMI *Z*-score (for each *Z*-score higher). Across subscales, ‘nice’ was the rationalisation with the highest level of agreement, while ‘necessary’ had the lowest (Table 2). Males agreed more strongly across all four subscales, when compared to females, with the greatest mean difference seen in the ‘nice’ subscale (−0·6; (95 % CI −0·9, −0·2)). There was no evidence of a relationship between age and meat consumption rationalisations. Those who lived in more deprived households were more likely to rationalise meat consumption as ‘nice’ compared to those of low deprivation. There was no evidence that BMI *Z*-score or weight status was related to the rationalisations that consuming meat is ‘natural’, ‘nice’ or ‘normal’, but a small tendency for those of higher BMI *Z*-score or weight status to agree that eating meat is not necessary.

**Table 2:** Demographic and anthropometric predictors of meat consumption rationalisation in non-vegetarians (*n* 337)

	Eating meat is natural		Eating meat is nice		Eating meat is necessary		Eating meat is normal	
	Mean difference	95 % CI	Mean difference	95 % CI	Mean difference	95 % CI	Mean difference	95 % CI
Mean	4·4		4·7		4·1		4·4	
sd *	1·2		1·3		1·5		0·9	
Sex, female compared to male	−0·4	−0·8, −0·1	−0·6	−0·9, −0·2	−0·3	−0·7, −0·02	−0·5	−0·7, −0·3
Age, years	−0·1	−0·2, 0·1	0·0	−0·2, 0·2	−0·1	−0·3, 0·1	0·0	−0·1, 0·1
Household deprivation†								
Low	Reference		Reference		Reference		Reference	
Medium	0·1	−0·2, 0·4	0·3	−0·02, 0·6	0·3	−0·03, 0·7	0·2	−0·1, 0·4
High	0·0	−0·3, 0·4	0·5	0·1, 0·9	0·2	−0·2, 0·6	0·2	−0·1, 0·5
BMI *Z*-score‡	−0·1	−0·2, 0·03	0·0	−0·2, 0·1	−0·2	−0·4, −0·05	0·0	−0·1, 0·1
Weight status‡								
Healthy weight	Reference		Reference		Reference		Reference	
Overweight	−0·3	−0·6, 0·03	−0·1	−0·5, 0·2	−0·4	−0·8, −0·01	−0·1	−0·3, 0·2
Obese	−0·1	−0·5, 0·4	0·3	−0·3, 0·8	−0·2	−0·7, 0·4	0·1	−0·2, 0·5

* Each subscale (natural, nice, necessary and normal) is scored using a seven-point scale from strongly disagree to strongly agree.

† Household deprivation measured using deciles of the NZDep2018 index, with Low: deciles 1–3; Medium: deciles 4–7; and High: deciles 8–10. Two participants in the non-vegetarian sample were missing these data.

‡ BMI *Z*-score calculated using the WHO growth charts. Overweight:BMI *Z*-score ≥1 & <2; Obese: BMI *Z*-score ≥2. 52 participants did not have anthropometric measures undertaken (*n* 45 non-vegetarians and *n* 7 vegetarians).

### Meat consumption rationalisation between vegetarians and non-vegetarians

**Fig. 1:**
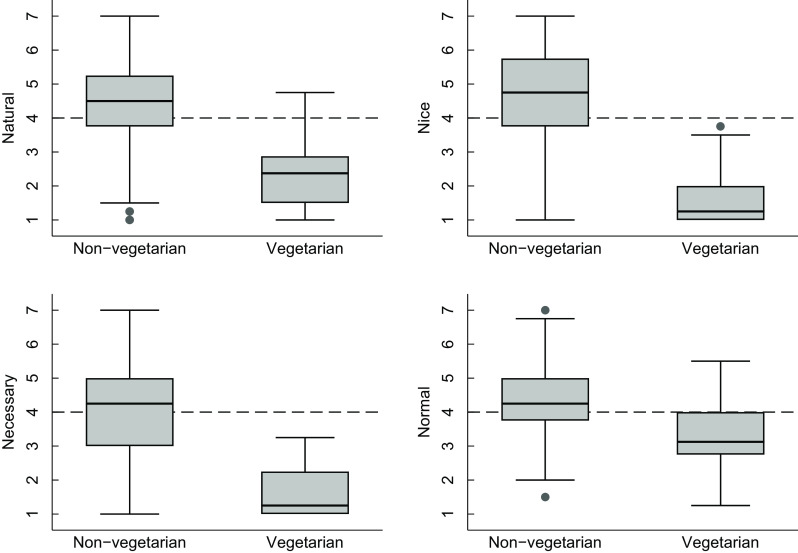
Box plots of meat rationalisation scores by self-identified vegetarian status (*n* 337 non-vegetarians and *n* 48 vegetarians). Each subscale is scored using a seven-point scale from strongly disagree to strongly agree about eating meat so that a score of 4 corresponded to ‘neither agree nor disagree’ (indicated by the dashed line)

Vegetarians disagreed with nearly all of the meat consumption rationalisations (Fig. 1). In non-vegetarians, 60·2 % agreed that eating meat was ‘natural’ (compared to 4·2 % of vegetarians), 64·7 % agreed that meat was ‘nice’ (compared to 0 % of vegetarians), 51·3 % agreed that meat was necessary (compared to 0 % of vegetarians) and 56·4 % agreed that eating meat was normal (compared to 14·6 % of vegetarians).

### Meat consumption rationalisation and meat consumption patterns

Table 3 reports mean differences (95 % CI) in the 4Ns subscale scores for low meat consumers and self-identified vegetarians compared to moderate/high meat consumers. Moderate/high meat consumers endorsed all 4N subscales more than low meat consumers and self-identifed vegetarians (Table 3). Self-identified vegetarians agreed to an even lesser extent that meat was natural, nice, necessary or normal, compared to the moderate/high meat consumers, with the greatest difference seen again in the ‘nice’ subscale (mean (sd) 4·8 (1·3) moderate/high *v*. 1·6 (0·8) self-identified vegetarian).

**Table 3:** Differences in meat consumption rationalisation by meat consumption patterns* (*n* 377)

	Mean	sd	Mean difference	95 % CI	*P*-value	Adjusted† mean difference	95 % CI	*P*-value
Natural								
Moderate/high meat consumers	4·5	1·1	Reference			Reference		
Low meat consumers	3·8	1·2	−0·7	−1·1, −04	<0·001	−0·6	−1·0, −0·3	0·001
Self-identified vegetarian	2·3	0·9	−2·2	−2·5, −1·8	<0·001	−2·1	−2·4, −1·7	<0·001
Nice								
Moderate/high meat consumers	4·8	1·3	Reference			Reference		
Low meat consumers	3·6	1·4	−1·2	−1·6, −0·8	<0·001	−1·1	−1·5, −0·7	<0·001
Self-identified vegetarian	1·6	0·8	−3·2	−3·6, −2·8	<0·001	−3·0	−3·4, −2·6	<0·001
Necessary								
Moderate/high meat consumers	4·2	1·4	Reference			Reference		
Low meat consumers	3·4	1·5	−0·8	−1·3, −0·4	<0·001	−0·8	−1·2, −0·3	0·001
Self-identified vegetarian	1·6	0·7	−2·5	−3·0, −2·1	<0·001	−2·4	−2·9, −2·0	<0·001
Normal								
Moderate/high meat consumers	4·4	0·9	Reference			Reference		
Low meat consumers	4·0	0·9	−0·5	−0·8, −0·2	0·003	−0·4	−0·7, −0·1	0·017
Self-identified vegetarian	3·3	1·0	−1·1	−1·4, −0·8	<0·001	−1·0	−1·2, −0·7	<0·001

* Meat consumption patterns determined using the Dietary Habits Questionnaire, which assessed *frequency* of meat consumption (processed meat, red meat, pork and poultry). Those who indicated that they consumed meat no more than five times a week were classified as ‘low meat consumers’ (*n* 39); those who indicated that they consumed meat more than five times a week were classified as ‘moderate/high meat consumers’ (*n* 290). Eight participants did not complete the Dietary Habits Questionnaire and were excluded from this analysis.

† Adjusted mean differences (95% CI) and *P*-values were adjusted for age, sex, and deprivation and accounted for school clusters.

### Meat consumption rationalisation and food choice motivations

On average, sensory appeal was the most important food choice motivation in non-vegetarians, closely followed by price, while religion was the least important in this sample of New Zealand adolescents (Table 4). Adolescent non-vegetarians whose food choice was motivated by convenience, sensory appeal, price and familiarity tended to agree more with all 4N subscales, indicated by the positive correlation coefficients, although correlations were weak (all less than 0·23). Whereas adolescents whose food choice was motivated by animal welfare and environmental concerns were less likely to agree with all 4N subscales, indicated by the negative correlation coefficients (again correlations were small, all less than 0·25).

**Table 4:** Correlations* between food choice motivations† and rationalisation of meat consumption in non-vegetarians (*n* 337)

	Mean	sd	Natural	Nice	Necessary	Normal
Health	2·6	0·7	−0·01	−0·14	0·03	−0·15
Mood	2·5	0·6	0·02	0·03	−0·01	−0·03
Convenience	2·6	0·6	0·17	0·17	0·12	0·17
Sensory appeal	2·8	0·6	0·23	0·16	0·15	0·15
Natural content	2·2	0·8	0·01	−0·10	−0·02	−0·12
Price	2·7	0·7	0·13	0·15	0·11	0·14
Weight control	2·3	0·8	0·00	−0·09	0·03	−0·04
Familiarity	2·2	0·7	0·17	0·17	0·16	0·11
Animal welfare	2·2	0·8	−0·12	−0·25	−0·14	−0·23
Environmental concerns	2·2	0·7	−0·12	−0·23	−0·14	−0·23
Religion	1·3	0·7	0·09	0·03	0·06	0·09

* Correlation coefficients are reported.

† Food choice motivations were measured using four-point scales from not at all important (1) to very important (4).

## Discussion

This is the first study in New Zealand to investigate the correlates of meat consumption rationalisation in male and female adolescents. Despite current recommendations and health messages surrounding the negative effects that high meat consumption can have^(4–6,9)^, over 50 % of non-vegetarian adolescents agreed that meat is ‘natural’, ‘nice’, ‘necessary’ and ‘normal’, while the low meat consumers and vegetarians were more likely to disagree with these rationalisations. Therefore, to be able to shift those with higher levels of meat consumption towards becoming low consumers, addressing these rationalisations will be important.

Moderate/high consumers of meat endorsed that meat was ‘nice’ the most (mean 4·8 (1·3)); a finding similar to that of Piazza *et al.*, (2015), where ‘nice’ was also endorsed the most in male and female adults (mean 5·0 and 4·5, respectively). Further to this, ‘sensory appeal’ was the most important food choice motivation in non-vegetarian adolescents. Adolescents have previously acknowledged that taste was of primary importance when they choose what to eat, while also revealing a general feeling that plant-based foods would not be as tasty because ‘meat has more flavour in it’ as well as it ‘looks like it’ll have more flavour’^(27)^. The taste and enjoyment of eating meat is one of the strongest barriers to transitioning to a plant-based diet for many, especially males^(28,29)^ with 23 % of adolescents in a South Australian study^(30)^ reporting they liked the taste of meat too much and had missed it on previous attempts at vegetarianism. The development and promotion of plant-based/meat-free meals that impart some of the same sensory qualities as meat may be needed to address this barrier. This trend is becoming evident in supermarkets and fast-food restaurants, where plant-based ‘meat’ options are increasing in availability^(31)^.

In contrast, the idea that meat consumption is ‘necessary’ was endorsed the least in non-vegetarian adolescents (mean 4·2 (1·4)), although still prevalent with 51 % agreeing to some extent. As ‘health’ was within the top four food choice motivations and was positively correlated to the ‘necessary’ subscale, this suggests that there may be some concern about potential health effects of eliminating meat from the diet. Concern over nutritional inadequacy has previously been raised in adults as a barrier to implementing a more plant-based diet^(8,28,32,33)^, specifically in regard to protein and Fe; therefore, it may be that this belief is held by adolescents also. Potential risks to health need to be well understood, especially for a population experiencing growth and puberty (as in adolescence)^(34,35)^, so that any dietary shift can be promoted safely and with confidence. At present, research investigating the health effects of low meat consumption in adolescents is limited^(36)^. Subsequent to this, reassurance that meat is not ‘necessary’ may differ dependent on sex, for example, males may want to consume meat for muscle development, whereas females may want to avoid Fe deficiency.

That meat consumption is ‘natural’ and ‘normal’ had similar results (4·5 (1·1) and 4·4 (0·9), respectively) in the moderate/high meat consumers; however, a greater mean difference between moderate/high and low consumers was observed for the ‘natural’ subscale (mean difference 0·7 ‘natural’ *v*. 0·4 ‘normal’). Meat eaters maintain that it is only natural to eat meat, with the perception that deriving nutrients from meat is part of being a human being^(14)^, a view Joy (2010) believes has come through socialisation, with customs and traditions shaping the habits of many. Furthermore, many consumers maintain meat-eating is an accepted and possibly expected social norm, part of human nature and biology and therefore normal^(3,14)^. Given this, although low meat eaters were less inclined to agree that eating meat is ‘natural’, their neutral stance on whether it is ‘normal’ or not somewhat agrees with Joy (2010) and Piazza *et al.* (2015). Yet this may also be due to the increased prevalence of plant-based or vegetarian diets in recent years^(37)^, which are also becoming part of the expected social norm.

Additional food choice motivations such as convenience, price and mood were also rated strongly. Convenience is a factor previously identified as a barrier to implementing a more plant-based diet in adults^(29)^, with many stating they did not know how to prepare plant-based meals^(8)^. Others believe it takes too long to prepare plant-based meals^(8)^ or that meat fits well with their usual dietary intake^(38)^. What foods are available in the home as well as who is preparing the meals will likely influence convenience as some adolescents have little say in this^(39)^. Plant-based or vegetarian foods have also been perceived as more expensive when compared to meat-based dishes^(8)^, and therefore cost is a barrier for some consumers. Thus, if plant-based food choices can be marketed as an affordable and convenient option, an increase in purchases may occur.

‘Familiarity’ tended to be positively associated with meat consumption rationalisations. Food neophobia has previously emerged as a barrier to plant-based food choice in adolescents^(27)^ as well as in adults^(29)^, with many expressing a reluctance to try new foods and preference for consuming familiar foods. The possibility of a growing willingness to try new foods could be motivated by adolescents’ increasing autonomy during this life stage or the influence peers can have on adolescent choices^(15,30,34,40)^.

Adolescents are known for their unhealthy food choices^(34)^ and although they have a basic understanding of the healthfulness of certain foods, they tend not to worry about the long-term health consequences of their food choices^(17,18,41)^. With current evidence suggesting family factors may positively influence the eating behaviour of adolescents, it is vital that education and interventions promoting the consumption of a more plant-based diet be extended to the families of adolescents^(42,43)^. In-school education exposing students and their families to a range of foods that are not meat-based alongside education of the nutritional and environmental benefits of a plant-based diet could have a widespread effect. With a focus on how plant-based meals can be prepared and how tasty and accessible plant-based options are, this could be a strategy to encourage positive health and planetary outcomes for generations to come.

While this study collected data from a range of adolescents, the results are limited by the non-representative sample. It is possible that subgroups of the population (e.g. different ethnic groups) might rationalise meat consumption in different ways and this should be considered in the interpretation of this data. Meat consumption was assessed by the Dietary Habits Questionnaire, which assessed frequency of consumption but did not provide quantitative estimates of consumption; however, this level of detail was not necessary for the objectives of this analysis. Despite these limitations, this study provides a set of results that can be used to inform future research and strategies to introduce a plant-based diet.

## Conclusion

Most adolescents rationalise their meat consumption because meat tastes good – a large proportion also think that eating meat is necessary, which may reflect concerns about nutritional inadequacies. It therefore seems likely that to facilitate adolescents to transition to a more plant-based eating pattern, we need to find/promote meat alternatives that taste good, are easy to prepare, are cost effective and provide nutritional adequacy.
